# Fungal Antagonism Assessment of Predatory Species and Producers Metabolites and Their Effectiveness on *Haemonchus contortus* Infective Larvae

**DOI:** 10.1155/2015/241582

**Published:** 2015-10-04

**Authors:** Manoel Eduardo Silva, Fabio Ribeiro Braga, Pedro Mendoza de Gives, Jair Millán-Orozco, Miguel Angel Mercado Uriostegui, Liliana Aguilar Marcelino, Filippe Elias de Freitas Soares, Andréia Luiza Araújo, Thainá Souza Vargas, Anderson Rocha Aguiar, Thiago Senna, Maria Gorete Rodrigues, Frederico Vieira Froes, Jackson Victor de Araújo

**Affiliations:** ^1^Departamento de Veterinária, Universidade Federal de Viçosa, Viçosa, MG, Brazil; ^2^Universidade Vila Velha, Vila Velha, ES, Brazil; ^3^Facultad de Ciencias Agropecuárias, UAEM, Avenida Universidad 1001, Col. Chamilpa, 62209 Cuernavaca, MOR, Mexico; ^4^Instituto Nacional de Investigaciones Forestales, Agricolas y Pecuarias INIFAP/CENID, Avenida Progreso No. 5, Colonia Barrio de Santa Catarina, 04010 Delegación Coyoacán, DF, Mexico; ^5^Departamento de Bioquímica e Biologia Molecular, Universidade Federal de Viçosa, Viçosa, MG, Brazil

## Abstract

The objective of this study was to assess antagonism of nematophagous fungi and species producers metabolites and their effectiveness on* Haemonchus contortus* infective larvae (L_3_). Assay A assesses the synergistic, additive, or antagonistic effect on the production of spores of fungal isolates of the species* Duddingtonia flagrans*,* Clonostachys rosea*,* Trichoderma esau*, and* Arthrobotrys musiformis*; Assay B evaluates in vitro the effect of intercropping of these isolates grown in 2% water-agar (2% WA) on L_3_ of* H. contortus*.* D. flagrans* (Assay A) produced 5.3 × 10^6^ spores and associated with* T. esau*,* A. musiformis*, or* C. rosea* reduced its production by 60.37, 45.28, and 49.05%, respectively.* T. esau* produced 7.9 × 10^7^ conidia and associated with* D. flagrans*,* A. musiformis*, or* C. rosea* reduced its production by 39.24, 82.27, and 96.96%, respectively.* A. musiformis* produced 7.3 × 10^9^ spores and associated with* D. flagrans*,* T. esau*, or* C. rosea* reduced its production by 99.98, 99.99, and 99.98%, respectively.* C. rosea* produced 7.3 × 10^8^ conidia and associated with* D. flagrans*,* T. esau*, or* A. musiformis* reduced its production by 95.20, 96.84, and 93.56%, respectively. These results show evidence of antagonism in the production of spores between predators fungi.

## 1. Introduction

Brazil has a herd of 212 million head of cattle and 171 million hectares of pastures that produce approximately 96% of Brazilian beef. On the other hand, gastrointestinal nematodes are a serious problem in ruminant production; once the animals have been exposed to high parasite loads they may succumb, especially younger individuals, which are more susceptible [1, 2].

The parasite-host relationship is characterized as a balanced relationship, being controlled by the intraspecified variability of host and parasite, allied to environmental factors. Epidemiologically whole herd raised under grazing conditions has some degree of gastrointestinal nematode infection, which results in a complex series of pathological events ranging between subclinical effects, production losses, and even death of the animal [3]. In this context, nematodes, especially the genus* Haemonchus*, are responsible for large economic losses in livestock. The conventional method for controlling such gastrointestinal parasites is to use synthetic anthelmintic drugs but they leave residues of the products in the treated animal, affect nontarget organisms, and select resistant strains of the parasites. Thus, use of nematophagous fungi, as an alternative control, has been constantly tested, with interesting results both in the field and under laboratory conditions [4].

A heterogeneous group of microfungi experts in capturing and using intestinal parasitic nematodes of domestic animals and phytonematodes as a source of nutrients are considered highly feasible and promising in environmental reduction of infective larvae [5, 6].* Duddingtonia flagrans* and* Arthrobotrys* spp. are fungi predators and the most studied and effective in the control of parasites of animals [4, 7].* Clonostachys* spp. and* Trichoderma* spp. are primarily used as biological controls of phytonematodes, but the diversity of the produced metabolites and adaptability to different environmental conditions give them the opportunity to be used in the biotechnology industry [7–10].

Although competition between microorganisms is very intense and severe making the introduction of new fungal species as biocontrol agents in certain environments practically impossible, it is necessary to evaluate the effect of combinations of fungi predators, opportunists, and ovicidal and metabolites producers and also abiotic factors, raising the possibility of establishment of fungi and potentiating the nematodes control [11–14]. Commercial spore production is usually performed in organic substrates or inert carriers, but several attempts to select substrates more productively and with low cost production have been carried out, especially with byproducts of agroindustry [15].

For use in biocontrol program as for survival and dissemination in environment condition of fungal spores is necessary [13] and when it comes to parasites animal control is important to select species and/or isolates capable of crossing the digestive tract and maintain the viability and predatory ability [16]. Thus, the objective of this study was to assess antagonism of nematophagous fungi and species producers metabolites and their effectiveness on L_3_ of* H. contortus*.

## 2. Materials and Methods

### 2.1. Organisms

Organisms were used as fungal isolates of* D. flagrans* (strain FTHO-8) [17],* Clonostachys rosea* (strain Yucatán, CICY-CONACYT),* Arthrobotrys musiformis*, and* Trichoderma esau* maintained on agar-water 2% (AA 2%) of mycology library of the National Institute for Forestry, Agricultural and Livestock, INIFAP, and* H. contortus* infective larvae (L_3_) (isolated Hueytamalco, Puebla) maintained by artificial infection in Pelibuey sheep in INIFAP facilities.

### 2.2. Spore Production

Two fragments of a previous crop in 2% WA of* D. flagrans*,* A. musiformis*,* C. rosea*, and* T. esau*, with approximately 4 mm^2^ being transferred alone or in association with other fungi to Petri dishes of 100 × 15 mm (06 replicates) containing 20 mL of 2% WhA culture medium, were maintained at room temperature and protected from light. The initial inoculum present in each fragment isolated from* D. flagrans*,* A. musiformis*,* C. rosea*, and* T. esau* was, respectively, 1.1 × 10^3^, 1.9 × 10^6^, 4.2 × 10^4^, and 7.1 × 10^5^ conidia and/or chlamydospores. After 7 days of cultivation 5 mL of distilled water was added on the plate surface and the spores were scraped with a spatula and stored in a sterile Falcon tubes. The volume was completed to 45 mL and 10 replicates were evaluated in a Neubauer chamber. Counting averages were extrapolated to the final volume of spores produced per plate.

### 2.3. “*In Vitro*” Test

Two fragments of a previous crop in 2% WA of* D. flagrans*,* A. musiformis*,* C. rosea*, and* T. esau*, with approximately 4 mm^2^ being transferred alone or associated with other fungi to Petri dishes of 600 × 15 mm (08 replicates) containing 10 mL of 2% WA culture medium, were maintained at room temperature and protected from light for 7 days. After cultivation, each Petri dish was inoculated with approximately 300 L_3_ of* H. contortus* obtained by the Baermann method from coprocultures of sheep faeces artificially infected. The Petri dishes were kept at room temperature and protected from light; in the seventh day of interaction between fungal isolates and nematodes, Baermann was taken from the agar contained in Petri plates for recovery of larvae not preyed upon.

### 2.4. Statistical Analysis

Assay A was designed in a factorial combination of fungal isolates × association of the same isolates (4 isolates, 6 associations, and 6 replicates) and Assay B was designed in a factorial combination of fungal isolates × nematode parasites (4 isolates, 6 associations, and 8 replicates) and one control group. The average number of produced spores or recovered larvae was subjected to analysis of variance and compared by Tukey test in 1% significance level using BioEstat 5.3 software.

## 3. Results and Discussion

**Figure 1:**
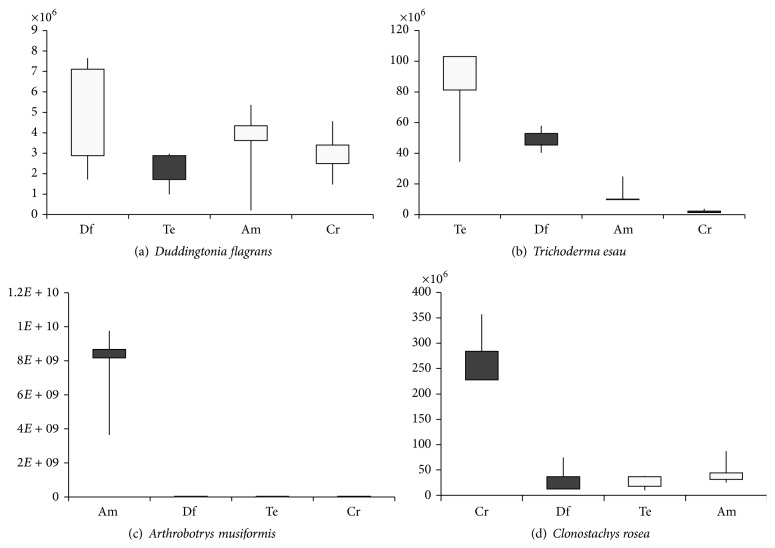
Mean and standard deviation of spores produced by different fungal isolates after 7 days on medium −2% wheat-agar (2% WhA).

The results observed in Table 1 show that, in the culture conditions described, the* D. flagrans* alone produced 5.3 × 10^6^ conidia/chlamydospores by Petri dish and when it was associated with* T. esau*,* A. musiformis*, or* C. rosea* the structure production was 2.1 × 10^6^, 2.9 × 10^6^, and 2.7 × 10^6^, respectively, showing a reduction of 60.37, 45.28, and 49.05%, respectively, in the spores production (Table 1 and Figure 1(a)).

The production of spores of* T. esau* alone, in the same conditions described, was 7.9 × 10^7^ conidia per plate and in association with* D. flagrans*,* A. musiformis*, or* C. rosea* was 4.8 × 10^7^, 1.4 × 10^7^, and 2.4 × 10^6^ structures, reducing at 39.24, 82.27, and 96.96%, respectively (Table 1 and Figure 1(b)).

*A. musiformis* alone produced 7.3 × 10^9^ conidia/chlamydospores per plate and in association with* D. flagrans*,* T. esau*, or* C. rosea* the reproductive structure production was 1.4 × 10^6^, 1.7 × 10^5^, and 1.4 × 10^6^, respectively, being reduced at 99.98, 99.99, and 99.98%, respectively (Table 1 and Figure 1(c)).

*C. rosea* alone reached a production of 2.8 × 10^8^ conidia per plate and in association with* D. flagrans*,* T. esau*, or* A. musiformis* the spores production was 3.5 × 10^7^, 2.3 × 10^7^, and 4.7 × 10^7^, being reduced at 95.20, 96.84, and 93.56%, respectively (Table 1 and Figure 1(d)).

The isolates,* A. musiformis* and* C. rosea* alone, showed the greatest production of reproductive structures per plate; on the other hand they also had the highest percentage of reduction in spores production when they were grown in association with other fungal isolates (Table 1).

In “*in vitro*” test the effectiveness of fungus predator* D. flagrans* was 100% against L_3_ of* H. contortus* in isolated culture (*p* < 0.01); the remaining 100% of efficacy cultivated in association with* A. musiformis* or* T. esau* was not antagonized by these fungi, but in association with* C. rosea* the predatory activity was reduced to 95.65% (Table 2).

The* A. musiformis* isolate grown in isolation preyed on 91.30% of larvae (*p* < 0.01) and associated with the fungus predator* D. flagrans* or metabolites producers,* T. esau* or* C. rosea*, showed additive predatory effect of 100, 100, and 95.65%, respectively (Table 2).

The fungi metabolites producers,* T. esau* and* C. rosea*, preyed on 82.60% of larvae grown in isolation (*p* < 0.01), but when they were grown in conjunction they showed strong antagonism, preying on 60.86% of larvae, not differing statistically from the control group (*p* > 0.05). In addition, these isolates showed additive effect by the presence of predators fungi* D. flagrans* or* A. musiformis*, preying on 100 and 95.65%, respectively, of L_3_ of* H. contortus* (Table 2).

### 3.1. Assay A: Evaluation of Spores Production

In this evaluation* D. flagrans* alone produced 5.3 × 10^6^ spores per Petri dish in AT 2% medium; Sagüés et al. [18] observed similar production with the addition of mesoinositol and wheat flour plus powder milk in Sabouraud Glucose Agar, SGA (5.1 × 10^7^ and 2 × 10^8^ chlamydospores, resp.), grown in a temperature of 27°C for 28 days, showing production potential of reproductive structures of* D. flagrans*. Hernández Mansilla et al. [19] obtained in biphasic system the production of 1 × 10^5^ chlamydospores/gram of* D. flagrans* in solid substrate (rice) after 30 days of culture at room temperature and demonstrated the efficacy of the spores on infective larvae of nematode of sheep. Boguś et al. [20] showed that the proteins of nematode are the best assailable nitrogen source for nematophagous fungi and then observed that nematodes homogenized induce the habit change from saprophyte to predator of* D. flagrans* in culture medium with a low concentration of carbon and nitrogen; these observations confirm the predatory potential of this species on infective larvae. The isolate of* D. flagrans* present a reduction of 60.37, 45.28, and 49.05% spores production when grown in association with* T. esau*,* A. musiformis*, or* C. rosea*, respectively, although Assis et al. [7] observed no difference in production of structures when that species was cultivated in association with the ovicidal fungus* Mucor circinelloides*; these observations demonstrated that isolated with different mechanisms of action may or not be antagonists in environmental conditions. In this case the antagonistic effect of metabolites producers fungi* T. esau* and* C. rosea* was more evident than that caused by predator fungus* A. musiformis* (Table 1).

The isolate of* A. musiformis* alone produced 7.3 × 10^9^ conidia/chlamydospores per Petri dish. When grown in association with* D. flagrans*,* T. esau*, or* C. rosea* the production of reproductive structures was, respectively, 1.4 × 10^6^, 1.7 × 10^5^, and 1.4 × 10^6^. This isolate shows decrease of the spores production by 99.98, 99.99, and 99.98%, respectively, demonstrating suffering greater antagonism by other fungal species (predators and/or producers of metabolites) (Table 1 and Figure 1(c)). This fact shows that this fungal species may not be a good competitor in the natural environment. Dias and Ferraz [21] observed the production of 2.58 × 10^6^ structures per Petri dish of spores of* A. musiformis* in YPSSA. Zhao et al. [22] showed that the disruption of* AoMls* gene of enzyme in the glyoxylate cycle, virulence factor in microbial pathogens from the nematode-trapping fungus* Arthrobotrys oligospora*, led to a significant reduction in conidiation and failure to utilize fatty acids and sodium acetate for growth, and its conidia were unable to germinate on minimal medium supplemented with sodium oleate.

The production of spores of* T. esau* alone was 7.9 × 10^7^ conidia per plate and associated with* D. flagrans*,* A. musiformis*, or* C. rosea* was 4.8 × 10^7^, 1.4 × 10^7^, and 2.4 × 10^6^ structures, respectively, reducing the spores production by 39.24, 82.27, and 96.96%, respectively, with statistically significant difference (*p* < 0.01), between the production alone and in association with other fungi (Table 1 and Figure 1(b)). These results support the data previously cited in which the fungal antagonism is exacerbated by producers species metabolites. This fact is highlighted by Hernández Mansilla et al. [19] who observed the antagonism of different species of* Trichoderma* on fungi phytopathogens that attack the pineapple.

The* C. rosea* isolate grown alone produced 2.8 × 10^8^ conidia per plate and in association with* D. flagrans*,* T. esau*, and* A. musiformis* produced 3.5 × 10^7^, 2.3 × 10^7^, and 4.7 × 10^7^ spores, respectively. The spores production of this isolate grown in association with* D. flagrans*,* T. esau*, or* A. musiformis* reduced spores production by 95.20, 96.84, and 93.56%, respectively, demonstrating suffering great antagonism from these species (Table 1 and Figure 1(d)). According to Sun et al. [23] the proportion of resistant spores of* C. rosea *67-1 increased to 17.4 and 15.5% in potato dextrose and rice meal media, respectively, in 8 days and the percentage of chlamydospores decreased rapidly with increased pH. Viccini et al. [24] observed that using rice as a substrate after 15 days of culture in plastic bottles and bags resulted in a production of 3.4 × 10^9^ and 1.1 × 10^8^ spores, respectively, per gram of dry matter, confirming the high productive potential of these fungal species.

Evaluating the production of reproductive structures presented in Table 1 demonstrates that all fungal isolates tested, producers metabolites and predators, exert strong antagonism when grown in conjunction, negatively influencing the production of spores in percentages ranging from 39.24 to 99.99% (*p* < 0.01); this fact exemplifies for the largest gathering and isolation of certain species in detriment to others in various environmental conditions.

### 3.2. Assay B: “*In Vitro*” Predation Test

In relation to “*in vitro*” test in this work it was observed that predators fungi* D. flagrans* and* A. musiformis* have the highest percentage of reduction of larvae compared with metabolites producers fungi. Santos et al. [25] assessed, in coprocultures performed using cattle faeces collected on the third day after the oral administration of large concentrations (200 grams' grain/animal) of isolate* A. musiformis*, 99% reduction in the number of L_3_ gastrointestinal nematodes. del Carmen Acevedo Ramírez et al. [26] isolated and identified isolates of* A. musiformis* in different regions of Mexico demonstrating their effectiveness* in vitro* against* H. contortus* infective larvae. Gutiérrez et al. [27] observed “*in vitro*” a reduction of 97% of L_3_ of* H. contortus* and 75% of (L_4_) histotrophic larvae by species,* A. musiformis*. Ojeda-Robertos et al. [28] observed that, after oral administration of chlamydospores of* D. flagrans* to naturally infected sheep, this isolate was able to reduce the number of larvae and eggs per gram of faeces in “*in vitro*” test confirming the predatory potential of these species on gastrointestinal helminths of veterinary and zootechnical importance.

The literature is scarce in studies that evaluate predatory activity of* T. esau* and* C. rosea* against gastrointestinal nematode infective larvae. In agricultural activities Ruano-Rosa et al. [29] observed that combinations of* T. atroviride* with strains* P. chlororaphis* and* P. pseudoalcaligenes* significantly improved the control of white root rot (WRR) caused by* R. necatrix* during the* in vitro* experiments, though a protective effect of* Trichoderma* and some bacteria has been observed in the control of WRR avocado when applied alone.

According to Baloyi et al. [9] isolate of* C. rosea *reduced nematode counts by 44% to 69.9%, in the faecal bioassay and in the water bioassay of 62.7% to 89.3% were observed. According to Ahmed et al. [30]* A. comosus* combined with* C. rosea* (AcCr) is better at controlling nematodes of sheep within a treatment which were paired and penned in individual paddocks than either Ac or Cr individually.* A. comosus* or AcCr reduced EPG and L_3_ counts on grass, but* C. rosea* only reduced L_3_. The daily feed of Merino sheep with 0.25, 0.5, and 1.0 g of* C. rosea *chlamydospores per kilogram BW reduced larval development (LD) time on day 70 of treatment by 33, 72, and 89%, respectively, in pastures. In the control group, LD was reduced by only 2.6% as the number of larvae per gram in faecal cultures [31].

On the other hand studies evaluating the use of fungal associations with different mechanisms of action are still emerging in the agriculture and animal sector. Tavela et al. [32] showed that the fungal isolates* Pochonia chlamydosporia*,* D. flagrans*, and* Monacrosporium thaumasium* were efficient in controlling horse cyathostomin under* in vitro* conditions, acting alone or in conjunction.

The findings in this study show the existence of fungal antagonism in the production of reproductive structures between species with potential use for control of gastrointestinal nematodes of domestic animals, especially among isolates predators and those producers of metabolites, although predatory reduction has not been observed in the effectiveness of this species. The biotic research in site for application nematophagous fungi with objective environmental control of gastrointestinal nematodes must be thoroughly investigated in order to obtain success in the parasite control program to be implemented.

## Figures and Tables

**Table 1:** Mean, standard deviation, and % of reduction of spores produced by different fungal isolates after 7 days on medium 2% wheat-agar (2% WhA).

*Duddingtonia flagrans*				
Isolate	*D. flagrans*	*T. esau*	*A. musiformis*	*C. rosea*
Conidia/chlamydospores	5.3 × 10^6^ ^a^	2.1 × 10^6^ ^b^	2.9 × 10^6^ ^b^	2.7 × 10^6^ ^b^
Standard deviation	(±2.1 × 10^6^)	(±7.1 × 10^5^)	(±1.5 × 10^6^)	(±8.7 × 10^5^)
% reduction	—	−60.37%	−45.28%	−49.05%

*Trichoderma esau*				

Isolate	*T. esau*	*D. flagrans*	*A. musiformis*	*C. rosea*
Conidia/chlamydospores	7.9 × 10^7^ ^a^	4.8 × 10^7^ ^ba^	1.4 × 10^7^ ^bb^	2.4 × 10^6^ ^bb^
Standard deviation	(±2 × 10^7^)	(±5.4 × 10^6^)	(±4.3 × 10^6^)	(±8.7 × 10^5^)
% reduction	—	−39.24%	−82.27%	−96.96%

*Arthrobotrys musiformis*				

Isolate	*A. musiformis*	*D. flagrans*	*T. esau*	*C. rosea*
Conidia/chlamydospores	7.3 × 10^9^ ^a^	1.4 × 10^6^ ^b^	1.7 × 10^5^ ^b^	1.4 × 10^6^ ^b^
Standard deviation	(±1.9 × 10^9^)	(±9.1 × 10^5^)	(±2.5 × 10^5^)	(±5.1 × 10^5^)
% reduction	—	−99.98%	−99.99%	−99.98%

*Clonostachys rosea*				

Isolate	*C. rosea*	*D. flagrans*	*T. esau*	*A. musiformis*
Conidia/chlamydospores	2.8 × 10^8^ ^a^	3.5 × 10^7^ ^b^	2.3 × 10^7^ ^b^	4.7 × 10^7^ ^b^
Standard deviation	(±3.9 × 10^7^)	(±2.1 × 10^7^)	(±9.2 × 10^6^)	(±2.1 × 10^7^)
% reduction	—	−95.20%	−96.84%	−93.56%

Different lowercase letters indicate existence of statistical difference (*p* < 0.01), Tukey test.

**Table 2:** Mean, standard deviation, and percentage of predation of *Haemonchus contortus* infective larvae recovered from Petri dishes by Baermann method after 7 days of interaction with different fungal species grown in conjunction or alone (Df = *Duddingtonia flagrans*, Am = *Arthrobotrys musiformis*, Te = *Trichoderma esau,* and Cr = *Clonostachys rosea*).

Parameters	Control	Df × Am	Df × Te	Df × Cr	Am × Te	Am × Cr	Te × Cr	Df	Am	Te	Cr
Mean	115^bb^	0^a^	0^a^	5^aa^	0^a^	5^aa^	45^ba^	0^a^	10^aa^	20^aa^	20^aa^
Deviation	±54.54	±0	±0	±13.23	±0	±13.23	±31.22	±0	±26.46	±28.28	±28.28
% predation	0%	100%	100%	95.65%	100%	95.65%	60.86%	100%	91.30%	82.60%	82.60%

Different lowercase letters indicate existence of statistical difference (*p* < 0.0001), Tukey test.

## References

[1] do Amarante A. F. T. (2011). Why is it important to correctly identify *Haemonchus* species?. *Revista Brasileira de Parasitologia Veterinaria*.

[2] da Silva M. E., Braga F. R., Borges L. A. (2014). Evaluation of the effectiveness of *Duddingtonia flagrans* and *Monacrosporium thaumasium* in the biological control of gastrointestinal nematodes in female bovines bred in the semiarid region. *Veterinary Research Communications*.

[3] Molento M. B., Almeida M., Guterres E., Roman J., Freitas F., Rocha M. (2005). Bezerras de corte infectadas naturalmente com parasitas gastintestinais—epidemiologia e tratamento e tratamento seletivo. *Archives of Veterinary Science*.

[4] Braga F. R., de Araújo J. V. (2014). Nematophagous fungi for biological control of gastrointestinal nematodes in domestic animals. *Applied Microbiology and Biotechnology*.

[5] Maciel A. S., Araújo J. V., Campos A. K. (2006). Viabilidade sobre larvas infectantes de *Ancylostoma* spp dos fungos nematófagos *Arthrobotrys robusta*, *Duddingtonia flagrans* e *Monacrosporium thaumasium* após esporulação em diferentes meios de cultura. *Revista Brasileira de Parasitologia Veterinária*.

[6] Mendoza-de-Gives P., Torres-Acosta F., Arias M. S., Paz-Silva A. (2012). Biotechnological use of fungi in the control of ruminant parasitic nematodes. *Fungi: Types, Environmental Impact and Role in Disease*.

[7] Assis R. C. L., Luns F. D., Araújo J. V. (2013). Comparison between the action of nematode predatory fungi *Duddingtonia flagrans* and *Monacrosporium thaumasium* in the biological control of bovine gastrointestinal nematodiasis in tropical southeastern Brazil. *Veterinary Parasitology*.

[8] Li J., Yang J., Huang X., Zhang K.-Q. (2006). Purification and characterization of an extracellular serine protease from *Clonostachys rosea* and its potential as a pathogenic factor. *Process Biochemistry*.

[9] Baloyi M. A., Laing M. D., Yobo K. S. (2011). Isolation and *in vitro* screening of *Bacillus thuringiensis* and *Clonostachys rosea* as biological control agents against sheep nematodes. *African Journal of Agricultural Research*.

[10] Martínez B., Infante D., Reyes Y. (2013). *Trichoderma* spp. y su función en el control de plagas en los cultivos. *Revista de Protección Vegetal*.

[11] Stirling G. R. (1991). *Biological Control of Plant Parasitic Nematodes: Progress, Problems and Prospects*.

[12] De leij F. A. A. M., Kerry B. R., Dennehy J. A. (1992). The effect of fungal application rate and nematode density on the effectiveness of *Verticillium chlamydosporium* as a biological control agent for *Meloidogyne incognita*. *Nematologica*.

[13] Lopes E. A., Ferraz S., Ferreira P. A. (2007). Potencial de isolados de fungos nematófagos no controle de *Meloidogyne javanica*. *Nematologia Brasileira*.

[14] Swe A., Li J., Zhang K. Q., Pointing S. B., Jeewon R., Hyde K. D. (2011). Nematode-trapping fungi. *Current Research in Environmental & Applied Mycology*.

[15] da Silva M. E., de Araújo J. V., Braga F. R., Soares F. E. D. F., Rodrigues D. S. (2012). Control of infective larvae of gastrointestinal nematodes in heifers using different isolates of *Nematophagous fungi*. *Revista Brasileira de Parasitologia Veterinaria*.

[16] Braga F. R., Carvalho R. O., Silva A. R. (2014). Predatory capability of the nematophagous fungus *Arthrobotrys robusta* preserved in silica gel on infecting larvae of *Haemonchus contortus*. *Tropical Animal Health and Production*.

[17] Llerandi-Juárez R. D., Mendoza-de Gives P. (1998). Resistance of chlamydospores of nematophagous fungi to the digestive processes of sheep in Mexico. *Journal of Helminthology*.

[18] Sagüés M. F., Fusé L. A., Iglesias L. E., Moreno F. C., Saumell C. A. (2013). Optimization of production of chlamydospores of the nematode-trapping fungus *Duddingtonia flagrans* in solid culture media. *Parasitology Research*.

[19] Hernández Mansilla A. A., García B. L. M., Álvarez C. R., González C. C., González Á. C. P., Mayea A. L. (2010). Control químico de patógenos fungosos en piña de vivero (I). *Fitosanidad*.

[20] Boguś M. I., Czygier M., Kȩdra E., Samborski J. (2005). In vitro assessment of the influence of nutrition and temperature on growing rates of five *Duddingtonia flagrans* isolates, their insecticidal properties and ability to impair *Heligmosomoides polygyrus* motility. *Experimental Parasitology*.

[21] Dias W. P., Ferraz S. (1992). Crescimento e esporulação de *Arthrobotrys* spp em diferentes substratos, meios de cultura, pH e níveis de temperatura. *Nematologia Brasileira*.

[22] Zhao X., Wang Y., Zhao Y., Huang Y., Zhang K.-Q., Yang J. (2014). Malate synthase gene AoMls in the nematode-trapping fungus *Arthrobotrys oligospora* contributes to conidiation, trap formation and pathogenicity. *Applied Microbiology and Biotechnology*.

[23] Sun M. H., Chen Y. M., Liu J. F., Li S. D., Ma G. Z. (2014). Effects of culture conditions on spore types of *Clonostachys rosea* 67-1 in submerged fermentation. *Letters in Applied Microbiology*.

[24] Viccini G., Mannich M., Capalbo D. M. F., Valdebenito-Sanhueza R., Mitchell D. A. (2007). Spore production in solid-state fermentation of rice by *Clonostachys rosea*, a biopesticide for gray mold of strawberries. *Process Biochemistry*.

[25] Santos C. P., Padilha T., Saumell C. A. Efficacy of *Arthrobotrys musiformis* in reducing infective larvae of trichostrongylid nematodes in fecal cultures after passage through bovine gastrointestinal tract.

[26] del Carmen Acevedo Ramírez P. M., Quiroz-Romero H., Valero-Coss R. O., Mendoza-de-Gives P., Gomez P. J. L. (2011). Nematophagous fungi from Mexico with activity against the sheep nematode *Haemonchus contortus*. *Revista Ibero-Latinoamericana de Parasitología*.

[27] Gutiérrez I. C. F., Mendoza-de-Gives P. M., Hernández E. L., López-Arellano M. E., Valero-Coss R. O., Velázquez V. M. H. (2011). Nematophagous fungi (Orbiliales) capturing, destroying and feeding on the histotrophic larvae of *Haemonchus contortus* (Nematoda: Trichostrongylidae). *Revista Mexicana de Micologia*.

[28] Ojeda-Robertos N. F., Torres-Acosta J. F. D. J., Aguilar-Caballero A. J. (2008). Assessing the efficacy of *Duddingtonia flagrans* chlamydospores per gram of faeces to control *Haemonchus contortus* larvae. *Veterinary Parasitology*.

[29] Ruano-Rosa D., Cazorla F. M., Bonilla N., Martín-Pérez R., De Vicente A., López-Herrera C. J. (2014). Biological control of avocado white root rot with combined applications of *Trichoderma* spp. and rhizobacteria. *European Journal of Plant Pathology*.

[30] Ahmed M., Laing M. D., Nsahlai I. V. (2014). A new control strategy for nematodes of sheep using chlamydospores of a fungus, *Clonostachys rosea f. rosea*, and an ethanolic extract of a plant, *Ananas comosus*. *Biocontrol Science and Technology*.

[31] Ahmed M., Laing M. D., Nsahlai I. V. (2014). Use of *Clonostachys rosea* against sheep nematodes developing in pastures. *Biocontrol Science and Technology*.

[32] Tavela A. O., Araújo J. V., Braga F. R. (2012). In vitro association of nematophagous fungi *Duddingtonia flagrans* (AC001), *Monacrosporium thaumasium* (NF34) and *Pochonia chlamydosporia* (VC1) to control horse cyathostomin (Nematoda: Strongylidae). *Biocontrol Science and Technology*.

